# Chemical angioplasty vs. balloon plus chemical angioplasty for delayed cerebral ischemia: a pilot study of PbtO_2_ outcomes

**DOI:** 10.1007/s00701-024-06066-2

**Published:** 2024-04-16

**Authors:** Eleanor M. Moncur, Claudia L. Craven, Selma Al-Ahmad, Bethany Jones, Fergus Robertson, Ugan Reddy, Ahmed K. Toma

**Affiliations:** 1 Victor Horsley Department of Neurosurgery, National Hospital for Neurology and Neurosurgery, Queen Square, London, WC1N 3BG UK; 2 Department of Brain Repair and Rehabilitation, UCL Queen Square Institute of Neurology, London, UK; 3 Department of Neurosurgery, Cambridge University Hospitals NHS Foundation Trust, Hills Road, Cambridge, CB2 0QQ UK; 4 Lysholm Department of Neuroradiology, National Hospital for Neurology and Neurosurgery, Queen Square, London, WC1N 3BG UK

**Keywords:** Delayed cerebral ischaemia, Vasospasm, Angioplasty, Brain tissue oxygen

## Abstract

**Background:**

Delayed cerebral ischaemia (DCI) is a major cause of morbidity and mortality after aneurysmal subarachnoid haemorrhage (aSAH). Chemical angioplasty (CA) and transluminal balloon angioplasty (TBA) are used to treat patients with refractory vasospasm causing DCI. Multi-modal monitoring including brain tissue oxygenation (PbtO_2_) is routinely used at this centre for early detection and management of DCI following aSAH. In this single-centre pilot study, we are comparing these two treatment modalities and their effects on PbtO_2_.

**Methods:**

Retrospective case series of patients with DCI who had PbtO_2_ monitoring as part of their multimodality monitoring and underwent either CA or TBA combined with CA. PbtO_2_ values were recorded from intra-parenchymal Raumedic NEUROVENT-PTO® probes. Data were continuously collected and downloaded as second-by-second data. Comparisons were made between pre-angioplasty PbtO_2_ and post-angioplasty PbtO_2_ median values (4 h before angioplasty, 4 h after and 12 h after).

**Results:**

There were immediate significant improvements in PbtO_2_ at the start of intervention in both groups. PbtO_2_ then increased by 13 mmHg in the CA group and 15 mmHg in the TBA plus CA group in the first 4 h post-intervention. This improvement in PbtO_2_ was sustained for the TBA plus CA group but not the CA group.

**Conclusion:**

Combined balloon plus chemical angioplasty results in more sustained improvement in brain tissue oxygenation compared with chemical angioplasty alone. Our findings suggest that PbtO_2_ is a useful tool for monitoring the response to angioplasty in vasospasm.

## Introduction

Aneurysmal subarachnoid haemorrhage (aSAH) is associated with high morbidity and mortality. Delayed cerebral ischaemia (DCI) is considered one of the most common causes of complications [9, 14].

Whilst vasospasm refers to angiographic evidence of arterial narrowing, DCI refers to the related neurological deterioration which is not attributable to hydrocephalus, seizures or medical causes [22]. Typical features of clinical deterioration due to DCI are the development of neurological deficits as a result of focal ischaemia, or a decrease in the level of consciousness as a result of global ischaemia [22]. DCI remains a poorly understood phenomenon and is a major predictor of poor outcomes in aSAH [2, 5]. When DCI is found in combination with evidence of arterial narrowing on angiographic imaging, this is referred to as ‘symptomatic vasospasm’.

Current management strategies for symptomatic vasospasm focus on intensive care medical management with augmented haemodynamic therapies.

Symptomatic vasospasm refractory to such measures can be treated with angioplasty as a rescue measure, using intra-arterial vasodilators (chemical angioplasty (CA)), transluminal balloon angioplasty (TBA) or a combination of both (TBA plus CA) [9, 19]. Traditionally, angioplasty of symptomatic cerebral vasospasm uses a mix of TBA for proximal vasospasm, followed by CA for distal vasospasm or for vessels deemed at risk of rupture if a balloon was to be used [10]. Angioplasty has been shown to achieve significant improvement when employed early after onset of neurological deficits; therefore, timely diagnosis is essential [1].

For patients who are sedated in intensive care, prompt diagnosis of DCI can be challenging. Parenchymal measurement of partial pressure of brain tissue oxygen (PbtO_2_) allows continuous objective real-time assessment of brain oxygen tension and therefore early identification of patients at risk of DCI [4]. Lower mean PbtO_2_ and longer periods of compromised cerebral oxygenation are associated with poorer outcomes and death in aSAH [15].

Multi-modal monitoring including PbtO_2_ is routinely used at this centre to assess and guide management in DCI. PbtO_2_ is also monitored during angioplasty to objectively determine when successful reperfusion has been established. At this centre, combined approach (TBA plus CA) is preferred unless TBA is too high risk.

In this pilot study, we aimed to compare the effects of CA alone versus TBA and CA, on PbtO_2_. We hypothesised that the PbtO_2_ responses would be more sustained in patients treated with a combined approach.

## Methods and materials

This study was performed as part of a retrospective service evaluation.

### Population

We included all patients with aSAH admitted to this single centre between 2018 and 2019, with suspected DCI and an intra-parenchymal Raumedic NEUROVENT-PTO® in situ as part of their multimodal monitoring throughout the angioplasty process. Any patients who had already undergone a previous angioplasty procedure were excluded in order to capture the effectiveness of TBA plus CA versus CA alone as an initial treatment. All patients had secured aneurysms; were ventilated, sedated and monitored in the neurocritical care unit (NCCU); and received daily nimodipine and transcranial Doppler ultrasound (TCD) from the day of admission.

### Placement of PbtO_2_ Bolts

The PbtO_2_ probe was inserted at the site that most likely would be affected by the vascular spasm as seen on either CTA or angiography (e.g. for anterior circulation vasospasm, the bolt could be placed slightly more medial, and for posterior circulation vasospasm the bolts were placed occipitally). This procedure was done at the bedside in the NCCU.

### Criteria for interventional angiography

Patients with persistently low PbtO_2_ (which was defined as less than 15 mmHg at the time of this study) underwent a local protocol of escalated medical management for DCI, including (a) temporary hyperoxia (for example, increase FiO_2_ to 100% for 5 min to check there is a response (and no wire issues); (b) optimising blood gas, ensuring airway does not require suctioning and keep PaCO_2_ 5.0–5.5 kPa, PaO_2_ 13–16 kPa); (c) ensuring ICP was managed optimally; (d) optimisation of haemoglobin (keeping levels above 100 g/L with blood transfusion); (e) treatment of pyrexia (commencing cooling with target temperature of 36 °C); (f) increasing the level of induced hypertension (in patients with secured aneurysms can be increased incrementally to 200–220 mmHg with maximum noradrenaline rare 0.8 mcg/kg/min); and (g) and finally a trial of hyperoxia with FiO_2_ increased to achieve a PaO_2_ greater than 18 Kpa and maximum FiO_2_ 60%. If PbtO_2_ did not improve (after 30 min) despite the above maximal medical management, a multidisciplinary discussion was undertaken regarding the value of interventional procedure for vasospasm. This was influenced by patient factors as well as findings on TCD and CT angiogram and in patients deemed appropriate, angiography followed by intervention (either CA or TBA plus CA) were performed directly.

### Intervention

All patients in this study underwent either CA (verapamil) or TBA plus CA as part of the standard clinical management in this centre. The treatment decision was made by the treating interventional radiologist. The general principles underlying this decision were that patients with proximal vasospasm amenable to balloon angioplasty underwent this procedure whilst those with more distal, or anatomically riskier vasculature, underwent chemical angioplasty alone. Data from the NEUROVENT-PTO® were continuously collected pre-, during and post-angioplasty and downloaded as second-by-second data.

### Comparison

Comparison between pre-angioplasty PbtO_2_ and post-angioplasty PbtO_2_ median values (4 h before angioplasty, 4 h after and 12 h after).

### Outcome

The recorded outcome measure was PbtO_2_ levels and how long they were sustained for after treatment.

### Angioplasty procedure

Angiographic procedures were performed under general anaesthesia via common femoral artery access. Bilateral internal carotid and, depending on the location of vasospasm, vertebral artery angiography was performed. Cerebral vasospasm was assessed by the interventional neuroradiologist. CA was performed using intra-arterial infusion of 10 mg of verapamil into each target artery over up to 30 min. Response to verapamil was determined angiographically. Where there was proximal vasospasm, systemic heparinisation was administered adapted to weight, and then TBA of the target vessel segment was performed. TBA was performed via 4- to 6-F catheters with Transform (Stryker) or Maverick balloons using Envoy guidewires. Response to TBA was again determined angiographically.

### Statistical analysis

All comparative tests were performed in GraphPad Prism version 10.0.1. Continuous data are presented as medians and ranges and categorical data are presented as counts. Continuous demographic data were compared using an unpaired *t*-test. Categorical data were compared using chi-square test for trend and Fisher’s exact test. Comparisons between PbtO_2_ results (in mmHg) for (1) from baseline to 12 h post procedure within a group and (2) between chemical versus chemical plus TBA were performed using paired and unpaired *t*-tests respectively. A *p*-value of less than 0.05 was considered significant.

## Results

### Demographics

A total of ten patients with PbtO_2_ monitors underwent angioplasty, of which six had intra-arterial verapamil only, and four patients underwent a combination of chemical and mechanical balloon angioplasty. All patients underwent treatment within the first 14 days of ictus (Table 1).

**Table 1:** CA vs. TBA plus CA demographics

	CA	TBA plus CA	Significance
Total	*n* = 6	*n* = 4	
Age	54.5 ± 3.3	50.5 ± 14.6	*p* = 0.52
M:F	2:4	3:1	*p* = 0.52
mFisher Grade (median)	3.5 (range 2–4)	3.5 (range 2–4)	*p* = 0.88
Aneurysm location*			
ICA	1	0	*p* > 0.99
PICA	1	0	*p* > 0.99
PCom	1	0	*p* > 0.99
MCA	2	2	*p* > 0.99
ACom	1	2	*p* = 0.5
PbtO_2_ Mean ± SD − 4 h before (mmHg)	14.6 ± 14.4	22.5 ± 8.5	*p* = 0.36
PbtO_2_ Mean ± SD 4 h after (mmHg)	27.8 ± 20.9	37.3 ± 7.5	*p* = 0.36
PbtO_2_ Mean ± SD 12 h after (mmHg)	20.6 ± 10.9	36.0 ± 4.1	***p* = 0.02**

^*^ICA internal carotid artery, *PICA* posterior inferior cerebellar artery, *Pcom* posterior communicating artery, *MCA* middle cerebral artery, *Acom* anterior communicating artery

### PbtO_2_ response to angioplasty

**Fig. 1:**
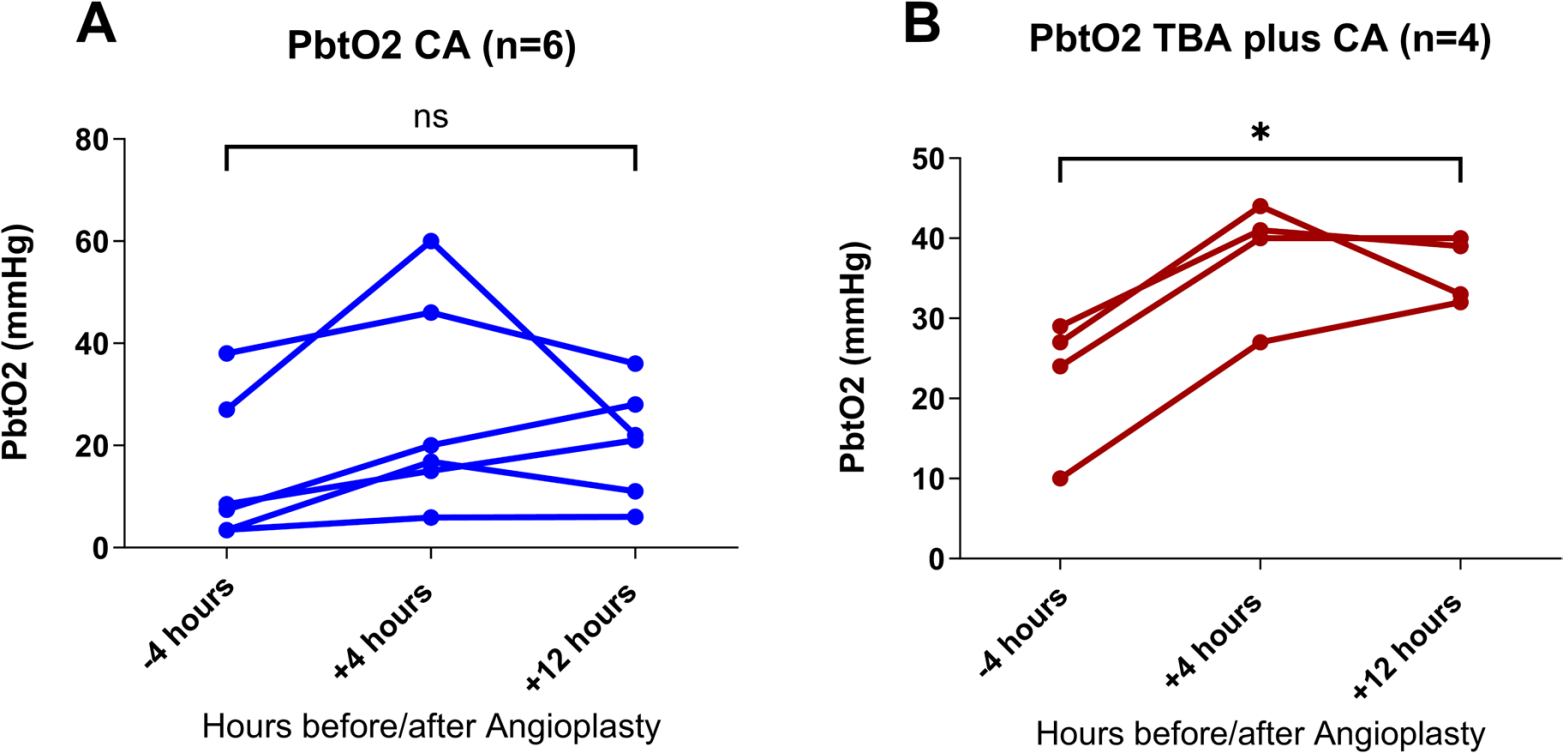
**A** Chemical angioplasty (*n* = 6) and** B** transluminal balloon angioplasty plus chemical angioplasty (*n* = 4). Brain tissue oxygenation before angioplasty (− 4 h) and after, at 4 h and 12 h (in mmHg)

For both interventions, there was an upward trend in PbtO_2_ of 13 mmHg and 15 mmHg for CA (Fig. 1A) and TBA plus CA (Fig. 1B) respectively, in the first 4 h post procedure. This improvement in PbtO_2_ was lost at 12 h in the CA group (Fig. 1A) whereas in the TBA plus CA group the effect was maintained at 12 h (Fig. 1B).

Within the CA group, PbtO_2_ levels increased by 6 mmHg from baseline to 12 h post angioplasty from 14.6 ± 14.4 to 20.7 ± 10.9 mmHg (mean ± SD) (Fig. 1A). This was not a significant increase (*p* = 0.18). Within the CA plus TBA group, PbtO_2_ levels increased by 13.5 mmHg from baseline to 12 h post angioplasty from 22.5 ± 8.5 to 36.0 ± 4.1 mmHg (mean ± SD) (Fig. 1B). This was a significant increase (*p* = 0.03).

**Fig. 2:**
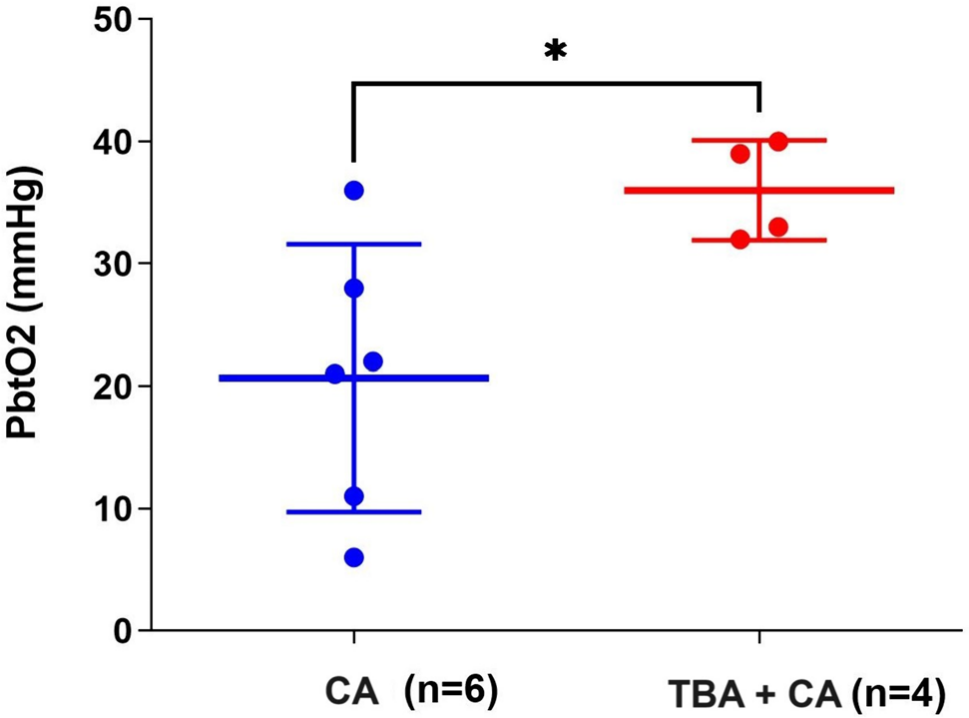
The difference in brain tissue oxygen (in mmHg) at 12 h following CA vs. TBA plus CA (*p* = 0.02)

The difference between the CA and TBA plus groups, at 12 h post procedure, was significantly different being 20 mmHg for those treated with CA, compared to 36 mmHg for those treated with TBA plus CA (*p* = 0.02) (Fig. 2).

One patient in the CA group developed an anterior circulation infarction post 14 days.

## Discussion

### Key findings

In this short pilot study, we compared the effect of chemical angioplasty versus a combined treatment of transluminal balloon plus chemical angioplasty on PbtO_2_ in patients with DCI post-aSAH. Both methods were effective within the first 4 h, but only the combined angioplasty caused a sustained improvement in PbtO_2_ at 12 h post-procedure. Our findings suggest that PbtO_2_ is useful as a neuromonitoring tool in this cohort and supports the routine use of PbtO_2_ monitoring in patients undergoing angioplasty.

### Chemical angioplasty

Few studies demonstrate a clear and sustained objective improvement of PbtO_2_ following chemical angioplasty treatment alone. In 1995, Leur and colleagues first demonstrated a brief improvement in brain oxygenation (using transcranial cerebral oximetry) following intra-arterial papaverine infusion for symptomatic cerebral vasospasm [11]. Other reports describe varying results, with either minimal benefit (no significant PbtO_2_ change 12 h post-CA), or clear sustained benefit (a minimum of 8 h or more significant increase in PbtO_2_) and good clinical outcomes[4, 21]. Near-infrared spectroscopy techniques also revealed inconsistent improvements in brain oxygenation after chemical angioplasty [12]. Interestingly, some studies found a transient (4–10 min) decrease in PbtO_2_ in patients receiving intra-arterial papaverine [20]. There was no mention of any beneficial effect after this but it was considered whether confounding factors such as a concomitant uncompensated decrease in intracranial pressure had contributed to the decrease in PbtO_2_ [20].

### Balloon angioplasty

Initial large-scale studies showed favourable outcomes when transluminal balloon angioplasty (TBA) is performed at an early stage [1, 17]. Santillan and colleagues described their positive experience over a 9-year period of treating distal cerebral vasospasm with TBA, concluding that it is both safe and effective, with 82.1% of their 28 patients showing good outcome (mRS < 2) [18]. This is also supported with evidence that balloon angioplasty can result in up to 5 days of improvement in brain oxygen tension (measured via Licox PtiO_2_ probe) [8]. Labeyrie et al. demonstrated in a large series of patients with cerebral vasospasm, that distal balloon angioplasty decreased the risk of DCI and recurrence of vasospasm compared with conventional angioplasty. Nonetheless, these findings did not correlate with a clinical benefit [10]. Schacht et al. provide data on TBA for cerebral vasospasm in their centre which corroborate the findings which suggest that TBA is a safe and effective treatment for vasospasm [19]. However, they warn of recurrent cerebral vasospasm as a common finding post-TBA, especially in those with moderate to poor angiographic response [19]. Whilst the majority of evidence supports TBA, some studies show minimal benefit of TBA alone in terms of clinical outcome [13, 16].

### Comparison of combined angioplasty and chemical angioplasty for DCI

Our findings are in agreement with existing evidence suggesting that combined angiography has a more sustained effect on brain oxygenation than chemical angiography alone.

The clinical outcome data supports a combined angioplasty approach. A literature review of 530 patients showed 62% improved clinically after TBA, compared to only 43% for CA alone (intra-arterial papaverine) [9]. Measures of brain oxygenation (transcranial Doppler velocities) were also significantly improved in the TBA group [9]. Furthermore, the clinical improvement in the CA group was only transient and there was a need for repeated interventions [9]. The same study found intra-arterial nicardipine to have more sustained improvement than papaverine.

Elliot et al. compared the effect of TBA with CA (a papaverine infusion) for the treatment of anterior circulation vasospasm post-SAH, using mean transcranial doppler (TCD) velocities pre- and post-treatment [6]. Both interventions demonstrated initial significant improvements; however, balloon angioplasty showed a more marked decrease (45% vs. 20%). Furthermore, in the papaverine group, velocities had deteriorated again by post-treatment day 2, necessitating re-treatment significantly more often than balloon angioplasty [6]. PbtO_2_ measurements have been shown to correlate with TCD findings, and so the fact that our data supports this study further validates the use of PbtO_2_ as a monitoring tool [3].

The comparison between CA (super selective intra-arterial papaverine infusion) and TBA has been analysed using continuous measurement of jugular bulb vein oxygen saturation (SvjO_2_) [7]. Nearly one third of patients who underwent TBA required additional papaverine in order to achieve normalisation of luminal diameter, suggesting that regardless of method used (TBA or CA), a combined approach is optimal [7].

### Strengths and limitations

Owing to the COVID-19 pandemic, this study was interrupted and therefore only low numbers are included. Nonetheless, the data is presented as a pilot study, to ensure the information gleaned is available.

Despite the low numbers, there is a clear objective difference between the CA and TBA plus CA group, in terms of sustained increase to PbtO_2_. Due to the PbtO_2_-based protocol-led management at this centre, all patients were subjected to the same standardised decision-making protocol, thus minimising the variability of management. Future studies could use PbtO_2_ as a target for angioplasty procedures.

One limitation of this study relates to its pragmatic design. Patients with vasospasm affecting narrow or friable vessels (which were sometimes more distal) were treated with only CA. Certainly, spasm affecting more distal vessels can be considered a different pathology to spasm affecting the circle of Willis vasculature. Therefore, the difference in PbtO_2_ response between TBA + CA and CA alone, may, in part, relate to the difference in proximal and distal vasospasm pathophysiology.

### Conclusion

In this study, balloon plus chemical angioplasty demonstrated greater and more sustained increases in brain tissue oxygen in patients with refractory vasospasm. Our findings suggest that PbtO_2_ is a useful tool for monitoring the response to angioplasty in vasospasm.

## Data Availability

All data were obtained from the secure electronic hospital record system National Hospital for Neurology and Neurosurgery, Queen Square, London, WC1N 3BG.

## Abbreviations

CTA: Computed tomography angiography
DCI: Delayed cerebral ischaemia
aSAH: Aneurysmal subarachnoid haemorrhage
PbtO_2_: Partial pressure of brain tissue oxygen
NCCU: Neurocritical care unit
TBA: Transluminal balloon angioplasty
TCD: Transcranial Doppler ultrasound

## References

[1] Bejjani GK, Bank WO, Olan WJ, Sekhar LN (1998) The efficacy and safety of angioplasty for cerebral vasospasm after subarachnoid hemorrhage. Neurosurgery 42(5):979–86 9588541 10.1097/00006123-199805000-00013

[2] Budohoski KP, Guilfoyle M, Helmy A, Huuskonen T, Czosnyka M, Kirollos R, Menon DK, Pickard JD, Kirkpatrick PJ (2014) The pathophysiology and treatment of delayed cerebral ischaemia following subarachnoid haemorrhage. J Neurol Neurosurg Psychiatry 85(12):1343–1353 24847164 10.1136/jnnp-2014-307711

[3] Craven CL, Sae-Huang M, Hoskote C, Watkins LD, Reddy U, Toma AK (2021) Relationship between brain tissue oxygen tension and transcranial Doppler ultrasonography. World Neurosurg 149:e942–e946 33513443 10.1016/j.wneu.2021.01.070

[4] Deshaies EM, Jacobsen W, Singla A, Li F, Gorji R (2012) Brain tissue oxygen monitoring to assess reperfusion after intra-arterial treatment of aneurysmal subarachnoid hemorrhage−induced cerebral vasospasm: a retrospective study. AJNR Am J Neuroradiol 33(7):1411–1415 22422178 10.3174/ajnr.A2971 PMC7965503

[5] Dodd WS, Laurent D, Dumont AS, Hasan DM, Jabbour PM, Starke RM, Hosaka K, Polifka AJ, Hoh BL, Chalouhi N (2021) Pathophysiology of delayed cerebral ischemia after subarachnoid hemorrhage: a review. J Am Heart Assoc 10(15):e021845 34325514 10.1161/JAHA.121.021845 PMC8475656

[6] Elliott JP, Newell DW, Lam DJ, Eskridge JM, Douville CM, Le Roux PD, Lewis DH, Mayberg MR, Grady MS, Winn HR (1998) Comparison of balloon angioplasty and papaverine infusion for the treatment of vasospasm following aneurysmal subarachnoid hemorrhage. J Neurosurg 88(2):277–284 9452236 10.3171/jns.1998.88.2.0277

[7] Fandino J, Kaku Y, Schuknecht B, Valavanis A, Yonekawa Y (1998) Improvement of cerebral oxygenation patterns and metabolic validation of superselective intraarterial infusion of papaverine for the treatment of cerebral vasospasm. J Neurosurg 89(1):93–100 9647178 10.3171/jns.1998.89.1.0093

[8] Hoelper BM, Hofmann E, Sporleder R, Soldner F, Behr R (2003) Transluminal balloon angioplasty improves brain tissue oxygenation and metabolism in severe vasospasm after aneurysmal subarachnoid hemorrhage: case report. Neurosurgery 52(4):970–4 12657196 10.1227/01.neu.0000053033.98317.a3

[9] Hoh BL, Ogilvy CS (2005) Endovascular treatment of cerebral vasospasm: transluminal balloon angioplasty, intra-arterial papaverine, and intra-arterial nicardipine. Neurosurg Clin N Am 16(3):501–16 15990041 10.1016/j.nec.2005.04.004

[10] Labeyrie M-A, Gaugain S, Boulouis G, Zetchi A, Brami J, Saint-Maurice J-P, Civelli V, Froelich S, Houdart E (2019) Distal balloon angioplasty of cerebral vasospasm decreases the risk of delayed cerebral infarction. AJNR Am J Neuroradiol 40(8):1342–1348 31320465 10.3174/ajnr.A6124 PMC7048486

[11] Luer MS, Dujovny M, Slavin KV, Hernandez-Avila G, Ausman JI (1995) Regional cerebral oxygen saturation during intra-arterial papaverine therapy for vasospasm: case report. Neurosurgery 36(5):1033–1036 7791970 10.1227/00006123-199505000-00024

[12] Meng L, Settecase F, Xiao J, Yu Z, Flexman AM, Higashida RT (2016) Initial clinical experience with near-infrared spectroscopy in assessing cerebral tissue oxygen saturation in cerebral vasospasm before and after intra-arterial verapamil injection. J Clin Neurosci 26:63–69 26765758 10.1016/j.jocn.2015.10.020

[13] Polin RS, Coenen VA, Hansen CA, Shin P, Baskaya MK, Nanda A, Kassell NF (2000) Efficacy of transluminal angioplasty for the management of symptomatic cerebral vasospasm following aneurysmal subarachnoid hemorrhage. J Neurosurg 92(2):284–290 10659016 10.3171/jns.2000.92.2.0284

[14] Rabinstein AA, Friedman JA, Weigand SD, McClelland RL, Fulgham JR, Manno EM, Atkinson JLD, Wijdicks EFM (2004) Predictors of cerebral infarction in aneurysmal subarachnoid hemorrhage. Stroke 35(8):1862–1866 15218156 10.1161/01.STR.0000133132.76983.8e

[15] Ramakrishna R, Stiefel M, Udoetuk J, Spiotta A, Levine JM, Kofke WA, Zager E, Yang W, Leroux P (2008) Brain oxygen tension and outcome in patients with aneurysmal subarachnoid hemorrhage. J Neurosurg 109(6):1075–1082 19035722 10.3171/JNS.2008.109.12.1075

[16] Rasmussen R, Bache S, Stavngaard T, Skjøth-Rasmussen J, Romner B (2015) Real-time changes in brain tissue oxygen during endovascular treatment of cerebral vasospasm. Acta Neurochir Suppl 120:183–186 25366621 10.1007/978-3-319-04981-6_31

[17] Rosenwasser RH, Armonda RA, Thomas JE, Benitez RP, Gannon PM, Harrop J (1999) Therapeutic modalities for the management of cerebral vasospasm: timing of endovascular options. Neurosurgery 44(5):975–9 10232530 10.1097/00006123-199905000-00022

[18] Santillan A, Knopman J, Zink W, Patsalides A, Gobin YP (2011) Transluminal balloon angioplasty for symptomatic distal vasospasm refractory to medical therapy in patients with aneurysmal subarachnoid hemorrhage. Neurosurgery 69(1):95–101 21368694 10.1227/NEU.0b013e31821424f9

[19] Schacht H, Küchler J, Boppel T, Leppert J, Ditz C, Schramm P, Neumann A (2020) Transluminal balloon angioplasty for cerebral vasospasm after spontaneous subarachnoid hemorrhage: a single-center experience. Clin Neurol Neurosurg 188:105590 31759310 10.1016/j.clineuro.2019.105590

[20] Stiefel MF, Spiotta AM, Udoetuk JD, Maloney-Wilensky E, Weigele JB, Hurst RW, LeRoux PD (2006) Intra-arterial papaverine used to treat cerebral vasospasm reduces brain oxygen. Neurocrit Care 4(2):113–118 16627898 10.1385/NCC:4:2:113

[21] Stuart RM, Helbok R, Kurtz P et al (2011) High-dose intra-arterial verapamil for the treatment of cerebral vasospasm after subarachnoid hemorrhage: prolonged effects on hemodynamic parameters and brain metabolism. Neurosurgery 68(2):337–45 21135735 10.1227/NEU.0b013e318201be47

[22] Vergouwen MDI, Vermeulen M, van Gijn J et al (2010) Definition of delayed cerebral ischemia after aneurysmal subarachnoid hemorrhage as an outcome event in clinical trials and observational studies: proposal of a multidisciplinary research group. Stroke 41(10):2391–2395 20798370 10.1161/STROKEAHA.110.589275

